# CHALLENGES IN THE COMMUNITY-DRIVEN RECRUITMENT OF BLACK OLDER ADULTS TO A HEALTH RESEARCH REGISTRY

**DOI:** 10.1093/geroni/igad104.0278

**Published:** 2023-12-21

**Authors:** Tam Perry, Kent Key, Elena Flores, Vanessa Rorai, Jamie Mitchell

**Affiliations:** Wayne State University, Detroit, Michigan, United States; Michigan State University, East Lansing, Michigan, United States; University of Michigan, Ann Arbor, Michigan, United States; Healthier Black Elders Center, Detroit, Michigan, United States; University of Michigan, Ann Arbor, Michigan, United States

## Abstract

Black older adults are underrepresented in health research. Community members representing diverse aging-focused organizations in Flint, Michigan desired more accessible health discoveries, and formed a Community Advisory Board (CAB) in partnership with three local universities. With a grant from the National Institute on Aging (NIH), the CAB sought to promote research engagement through free community health programming and launching a Participant Research Pool (PRP) to connect Black older adults with non-invasive IRB-approved health research at participating universities. Despite extensive recruitment efforts by this academic-community partnership, including monthly virtual health seminars, a new website, tailored print and video promotional materials, a minority aging newsletter, and a community research symposium, the PRP remains severely under-enrolled after a year. An evaluation of recruitment strategies revealed a need for more frequent in-person programming, leveraging the social and professional networks of CAB members, increased intentionality in community partnerships, and more intensive and traditional modes of advertising to reach and recruit the target population. Lessons indicate that Black older adults in the community trust this research endeavor but require tailored and consistent outreach to engage with the participant research pool.

